# Effects of breastfeeding training programmes for midwives on breastfeeding outcomes: a systematic review and meta-analysis

**DOI:** 10.1186/s12884-023-05540-6

**Published:** 2023-04-18

**Authors:** Tianci Wang, Meimei Shang, Ka Ming Chow

**Affiliations:** 1 The First Affiliated Hospital, Sun Yat-Sen University, Guangzhou, China; 2 The Nethersole School of Nursing, Faculty of Medicine, The Chinese University of Hong Kong, Shatin, Hong Kong SAR; 3 Shandong Cancer Hospital and Institute, Shandong First Medical University, Jinan, China

**Keywords:** Breastfeeding, Midwives, Systematic review, Meta-analysis

## Abstract

**Background:**

Appropriate breastfeeding training for midwives is necessary to enhance their knowledge, attitude, and practice (KAP). However, evidence surrounding the effects of midwife breastfeeding training programmes is insufficient to draw a conclusion of its effectiveness on breastfeeding initiation, duration, and rates.

**Objective:**

The aim of this systematic review was to identify, summarise, and critically analyse the available literature to evaluate the effects of midwife breastfeeding training programmes on the midwives’ KAP towards breastfeeding and breastfeeding initiation, duration and rates among postnatal mothers.

**Methods:**

Nine English and six Chinese databases were searched with relevant key words. The methodological quality of the included studies were assessed by two reviewers independently using the Joanna Briggs Institute critical appraisal checklists.

**Results:**

Nine English and one Chinese articles were included in this review. Five articles investigating midwives’ KAP towards breastfeeding reported positive results (*p* < 0.05). The meta-analysis revealed that breastfeeding training programmes significantly improved midwives’ breastfeeding-related knowledge and skills (standardised mean difference = 1.33; 95% confidence interval, 0.98 to 1.68; *p* < 0.01; I^2^ = 36%), as well as their attitude towards breastfeeding (*p* < 0.05). An additional five articles measured the effects of breastfeeding training programmes on the initiation, duration, and rates of breastfeeding among postnatal mothers. Following the implementation of a breastfeeding training programme for midwives, mothers had significantly longer durations of exclusive breastfeeding (*p* < 0.05), fewer breastfeeding challenges (*p* < 0.05) (e.g. breast milk insufficiency), and higher satisfaction with breastfeeding counselling (*p *< 0.01), and fewer infants received breast milk substitutes in their first week of life without medical reasons (*p* < 0.05) in the intervention group compared with the control group. However, no significant effects were seen on the initiation and rates of breastfeeding after implementation of the programmes.

**Conclusions:**

This systematic review has demonstrated that midwife breastfeeding training programmes could improve midwives’ KAP towards breastfeeding. However, the breastfeeding training programmes had limited effects on breastfeeding initiation and rates. We suggest that future breastfeeding training programme should incorporate counselling skills alongside breastfeeding knowledge and skills training.

**Review registration:**

This systematic review has been registered in the International prospective register of systematic reviews (PROSPERO) (ID: CRD42022260216).

## Introduction

Breastfeeding is a topic of global attention. It is considered as the best way to feed a baby and has been shown to have substantial short- and long-term benefits forboth mothers and infants [1–6]. In view of the beneficial effects of breastfeeding, the World Health Organisation (WHO)/ United Nations Children’s Fund (UNICEF) Global Strategy on Infant and Young Child Feeding specifically recommended that governments protect, promote, and support breastfeeding [7]. The World Health Assembly has also developed a ‘comprehensive plan for mother, infant and child nutrition’ with the goal of increasing the rate of exclusive breastfeeding during the first six months to at least 50% by 2025. In mainland China, the Chinese State Council stated in its Programme for the Development of Children in China (2011–2020) that the goal of having 50% of infants breastfeed exclusively during their first six months should be reached by year of 2020 [8].

Despite extensive promotion of breastfeeding and related policy, breastfeeding rates remain low. In 2016, UNICEF reported that fewer than half of the babies (43%) worldwide were breastfed within the first hour of life and that only 41% of the infants were exclusively breastfed within their first six months in 2018 [9, 10]. In 2017 in mainland China, according to UNICEF’s Global Breastfeeding Scorecard, only 29% of the infants were breastfed within the first hour of birth, 21% were exclusively breastfed within their first six months, and only 24% and 7% continued to be breastfed until one or two years of age, respectively [11]. In comparison, in Asia (including China), the rate of breastfeeding initiation within the first hour of birth was 68%, and the rate of exclusive breastfeeding during the first six months of life was 26% [11].

The ‘Ten Steps to Successful Breastfeeding’ is recommended as a key component of the WHO and UNICEF Baby-friendly Hospital Initiative (BFHI), which has been widely implemented and has proven to be an efficient intervention to improve long-, intermediate-, and short-term breastfeeding outcomes worldwide [12–14]. Step two of the ‘Ten Steps to Successful Breastfeeding’ advises training healthcare staff in the knowledge and skills necessary to implement breastfeeding policy. Midwives, who provide nursing care for mothers throughout pregnancy and childbirth, play an important role in promoting breastfeeding and supporting postnatal mothers, with steps two to eight of the ‘Ten Steps to Successful Breastfeeding’ relating specifically to midwives.

However, a literature review revealed that health professionals find providing breastfeeding support challenging because they often do not have the necessary practical skills, and in most cases, professionals are not instructed about in how to promote breastfeeding [15]. Indeed, inadequate training in how to help mothers breastfeed their infants has been identified as a major factor contributing to inefficiency in professional practice and undermining breastfeeding [16, 17]. Therefore, to act as effective breastfeeding facilitators, appropriate breastfeeding training for midwives is necessary to enhance their breastfeeding support skills.

Prior systematic reviews of breastfeeding training programmes have focused on all health professionals (with or without midwives) who support breastfeeding mothers [18–20]. The targeted populations included nurses, midwives, doctors, and home visitors. As the breastfeeding knowledge and skills of various professional groups may differ, their training needs and the effects of such training can also vary. No systematic review has explored the effects of breastfeeding training programmes specifically targeted at midwives. Moreover, two systematic reviews only included a limited number of studies, four and six, respectively [18, 20]. The review by de Jesus et al. was conducted seven years ago and also needs to be updated [19]. Moreover, all previous reviews have primarily evaluated knowledge, attitude and practice (KAP) towards breastfeeding [18–20]. As a result, there is a lack of reviews exploring the impact of breastfeeding training programmes on breastfeeding initiation, duration, and rates. Therefore, it is necessary to conduct a review of breastfeeding training programmes specifically designed for midwives to identify their effects and identify areas for improvement in their design.

This systematic review has been registered in the International prospective register of systematic reviews (PROSPERO) (ID: CRD42022260216).

## Objectives

The objective of this systematic review was to identify, summarise, and critically appraise the evidence surrounding the effects of breastfeeding training programmes for midwives on the primary outcome of midwives’ KAP towards breastfeeding, and the secondary outcomes of breastfeeding initiation, duration, and rates among postnatal mothers. This study also aimed to identify areas for improvement in the design of breastfeeding training programmes for midwives.

## Methods

### Search strategy

A systematic search was conducted of nine English databases including PubMed, Embase, Web of Science, the British Nursing Index, ScienceDirect, Educational Resources Information Centre (ERIC), Cochrane Library, Global Health, and Scopus; and six Chinese databases including WanFang Data, China National Knowledge Infrastructure (CNKI), Weipu Chinese Science and Technology Journal Database, Chinese Biomedical Literature Database (CBM), Chinese Medical Current Contents (CMCC), and Hong Kong Index to Chinese Periodicals (HKInChiP). The keywords used for the searches were ‘training’, ‘training programme’, ‘course’ or ‘education’, and ‘midwives’ or ‘professionals’. The equivalent words in Chinese were searched in the Chinese databases. Databases were searched using the Medical Subject Heading (MeSH) term ‘breastfeeding’ (i.e. expanded to include all sub-terms) combined with the text word searches to obtain the final set of articles.

The search was conducted in December 2020. All records were imported into EndNote X9, and duplicates were removed. Titles and abstracts were then screened according to the study selection criteria. If the abstract met the inclusion criteria, the full text was further reviewed. The cited references and bibliographies of these articles were screened to further identify relevant studies that were not retrieved through the database search. No restrictions were applied to the searches in terms of publication year, language, country, or region. All databases were searched from their inception.

### Inclusion and exclusion criteria

Inclusion criteria for the review were as follows: (1) randomised controlled trials (RCTs) or quasi-experimental studies that examined the effects of breastfeeding training programmes for midwives; (2) the main target population of the training programme included midwives (measuring the primary outcomes of KAP towards breastfeeding among midwives) or mothers cared for by midwives (examining the secondary outcomes of breastfeeding initiation, duration and/or rates) (3) the intervention focused on any breastfeeding-related training involving midwives; (4) comparisons made against midwives who were not involved in the breastfeeding training programme.

Exclusion criteria for the review were as follows: (1) editorials, reflective studies, observational studies (e.g. cohort, cross-sectional, or case–control studies), qualitative methodology studies, and review; (2) breastfeeding training programmes not involving midwives; (3) training programmes not focused on breastfeeding (e.g. children’s nutrition, communication skills, or consultation skills), any kind of self-learning programme, or provision of learning materials alone (e.g. DVDs, educational videos, media learning resources, or other kinds of materials) to participants; (4) lacking a control group, and (5) measuring outcomes for specific populations such as preterm infants.

### Screening, data extraction, analysis and quality evaluation

The methodological quality assessment using the Joanna Briggs Institute (JBI) critical appraisal checklists, and data extraction of the included studies were conducted by two reviewers independently [21]. Any disagreements between the reviewers were resolved by discussion, and referral to a third reviewer in cases of unresolved doubts.

A structured extraction form was used to extract and summarise key information from the included studies, including: (1) title, journal, author(s), country, language, and year of publication; (2) methodological characteristics (study design, location, setting, trial period, and sample size); (3) participant characteristics (demographics and inclusion/exclusion criteria); (4) characteristics of intervention (theoretical framework, duration, content, materials, and formats); (5) outcome assessment (evaluation method (e.g. questionnaire, interview, observation, medical record review, or a standardised tool), source of data (e.g. mothers, healthcare providers, or medical records), timing of data collection); and (6) results. If any details of a study were unclear, the corresponding author of the study was contacted to retrieve the information.

As this review included both RCTs and quasi-experimental studies, we analysed the corresponding data separately for different study designs. Based on study design and outcome measurements, the results from comparable studies were pooled in a statistical meta-analysis using Review Manager 5.4. As the scales used in each study were different, the standardised mean difference (SMD) and 95% confidence interval (CI) were calculated for continuous data [22]. Random-effects meta-analysis was then carried out. The I^2^index was calculated to assess heterogeneity in the meta-analysis [23]. According to Higgins, Thompson, Deeks, and Altman I^2^values of 25%, 50%, and 75% represent low, moderate, and high levels of heterogeneity, respectively [24]. If studies had a heterogeneous design and were not suitable for combining with other studies outcomes, or I^2^ > 50%, the findings were reported in a narrative format.

## Results

### Study selection

**Fig. 1:**
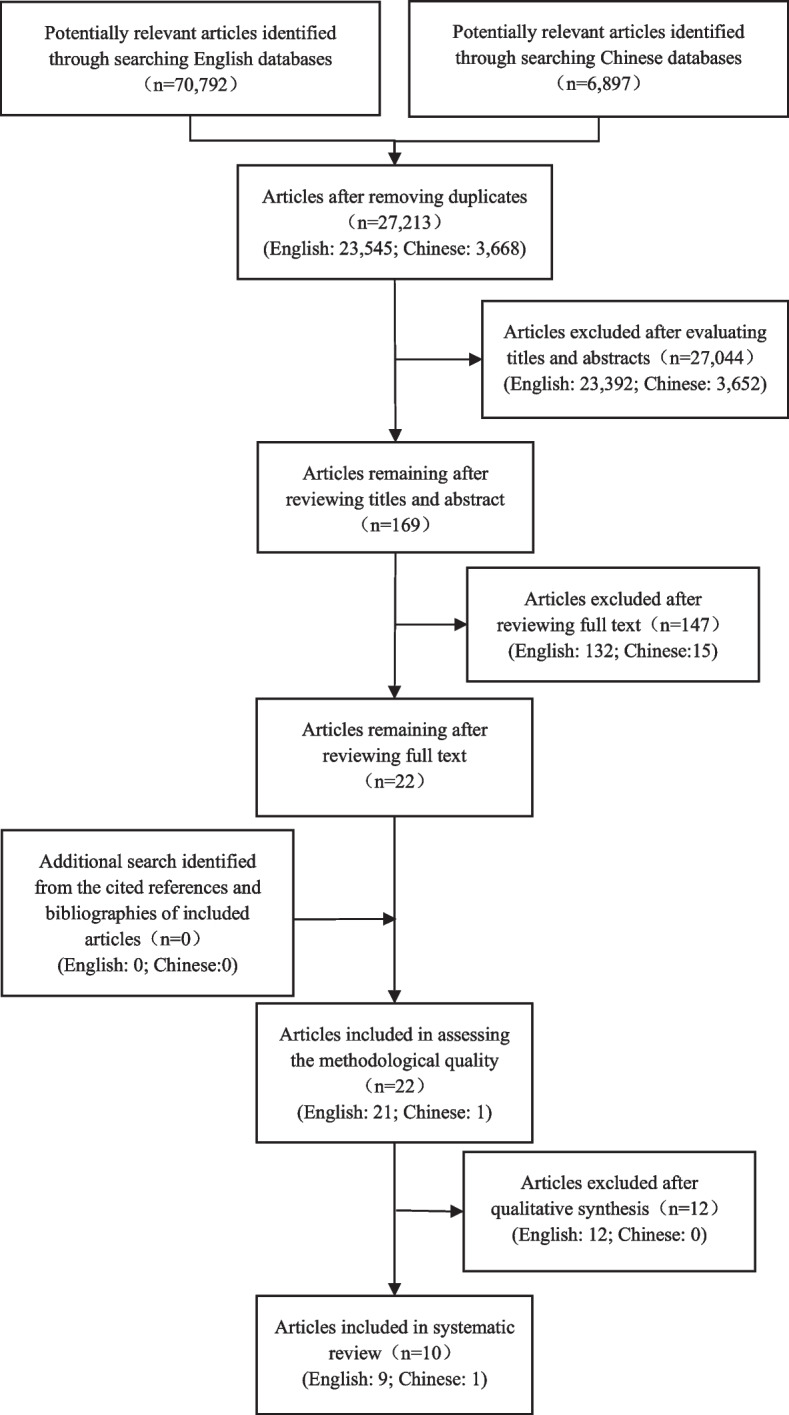
PRISMA flowchart of study retrieval and selection

In total, 70,792 English and 6,897 Chinese citations were identified using the search strategy. After removing duplicates, 23,545 English and 3,668 Chinese articles remained. However, most of these studies examined breastfeeding training programmes delivered by healthcare professionals to postnatal mothers, rather than those delivered to healthcare providers. Therefore, after reviewing the titles and abstracts, only 153 English and 16 Chinese articles were remained. Next, 132 English and 15 Chinese articles were further excluded after reviewing the full texts for the following reasons: (1) target audience did not include midwives (45 articles); (2) intervention did not focus on breastfeeding training (seven articles); (3) intervention was self-delivered or only provided learning resources to healthcare professionals (21 articles); (4) study design was not an RCT or a quasi-experimental study (63 articles); (5) study was not published in English or Chinese (six articles); (6) study did not measure the target outcomes (five articles). No relevant articles were retrieved from the reference list and bibliographies of the articles read in full. The remaining 21 English and one Chinese article were further assessed for eligibility and methodological quality and 12 English articles were removed based on the quality evaluation. Finally, nine English articles and one Chinese article, involving eight studies, as three articles reported the same study but with respect to different outcomes, were included in this systematic review. A PRISMA flowchart of study retrieval and selection process is presented in Fig. 1.

### Description of studies

The characteristics of the included studies are presented in Tables 1 and 2.

**Table 1:** Characteristics of studies measuring the primary outcome of midwives’ KAP towards breastfeeding (*n* = 5)

Author (year); country	Study design, sample size, and characteristics	Follow-up schedule	Intervention (breastfeeding training programme) and control groups	Outcome(s) and measure(s)	Results
Ekström et al.(2005); Sweden [25]	Longitudinal RCT. 28 midwives and 53 postnatal nurses During the follow-up period, 26 participants dropped out, resulting in a total response rate of 75.0% midwives and 64.2% nurses	Baseline and 1 year post-training	Intervention group: Programme: a process-oriented training programme Format: lectures and discussion Content: lectures on breastfeeding management and promotion, including discussions about counselling skills, and attitudes, and reflections on personal breastfeeding experiences Duration: 7 days Control group: No training	Attitudes towards breastfeeding: a validated self-designed breastfeeding attitudes instrument	• Breastfeeding training significantly improved attitudes towards breastfeeding of both midwives and postnatal nurses (*p* < 0.05) • Attitudes towards breastfeeding tended to be stable over 1 year, with only the regulating dimension scores decreasing slightly
Moran et al. (2000); UK [26]	Quasi-experimental design Intervention group: 15 midwives Control group: 13 midwives	No follow-up (data were only collected once after training)	Intervention group: Programme: the 20-hourWHO/ UNICEF breastfeeding management course Content: the basic knowledge, skills and attitudes required to teach and support breastfeeding women Duration: 20 h (generally taught over 3 separate days within a 3- to 6-week period) Provider: midwives and health visitors employed by UNICEF as part of the UNICEF UK Baby Friendly Initiative Materials: A comprehensive and fully referenced workbook was provided for each participant Control group: No training	Knowledge and skills: Breastfeeding Support Skills Tool (BeSST)	• Significantly increased breastfeeding knowledge and skills were seen in the intervention group compared with the control group after the breastfeeding training programme (*p* < 0.01)
Law et al. (2007); UK [27]	Quasi-experimental design Intervention group: 108 midwives Control group: 27 student midwives	Baseline and immediately after training	Intervention group: Programme: a breastfeeding training workshop Formats: lectures and role-play exercises Content: effective positioning and attachment and the use of hands-off teaching methods Duration: 4 h over 1 session Control group: No training	Knowledge and skills: BeSST	• Compared with baseline data, the total BeSST score increased significantly (*p* < 0.01) among participants in the intervention group after training • Compared with the control group, the total BeSST score was significantly higher in the intervention group (*p* < 0.01) after training, indicating the breastfeeding training programme enhanced midwives’ knowledge and skills
Wang et al. (2012); Taiwan [28]	Quasi-experimental design Intervention group: 30 midwives and nurses Control group: 30 midwives and nurses	Baseline and immediately after training	Intervention group: Programme: breastfeeding courses Content: the importance of breastfeeding, breastfeeding assessment and support, common problems, and breastfeeding initiation Duration: 2 h per week for 4 weeks Providers: breastfeeding teachers from the National Health Bureau, Department of Health, Executive Yuan Materials: the National Health Bureau, Department of Health, Executive Yuan (2005) Guidelines for Breastfeeding Teaching Materials in Taiwan and Breastfeeding Question and Answer Manual were used Control group: No training	Knowledge and skills: The Breastfeeding Knowledge Scale Attitudes towards breastfeeding: The Breastfeeding Attitude Scale	• After training, breastfeeding knowledge and skills scores were significantly higher in the intervention group than in the control group (*p* < 0.01) • After training, attitudes toward breastfeeding scores were significantly better in the intervention group than in the control group (*p* < 0.05)
Al-Nuaimi et al. (2019); USA [29]	Quasi-experimental design Intervention group: 42 midwives and nurses Control group: 40 midwives and nurses	Baseline and 2 weeks after training	Intervention group: Programme: An educational workshop Content: Anatomy and physiology of the breast; Physiology of milk production and hormonal physiology of breastfeeding; communication skills and building confidence with breastfeeding mothers; breastfeeding teaching methods; recommendations for healthy nutrition during breastfeeding; benefits of breastfeeding for mothers, infants and society; breastfeeding contraindications; common problems encountered by breastfeeding mothers; medications, or medical conditions that prevent or delay breastfeeding; appropriate positions for breastfeeding Duration: 2 h Materials: Based on up-to-date evidence, including recommendations from the WHO (2019) and the National Institute for Health and Care Excellence (NICE) (2015), 2 educational materials were developed, addressing the importance of breastfeeding initiation and child growth and development from birth to 5 years of age Control group: Provided with a 2-h workshop on child growth and development from birth to 5 years of age	Knowledge and skills: 2 validated questionnaires developed by the American Academy of Pediatrics Attitude towards breastfeeding: A 7-item breastfeeding attitude questionnaire	• After the educational workshop, knowledge and skills were significantly improved in the intervention group compared with the control group (*p* < 0.01) • After training, the intervention group had higher positive attitude scores than the control group (*p* < 0.01)

**Table 2:** Characteristics of studies measuring the secondary outcomes of breastfeeding initiation, duration, and rates (*n* = 5, with 3 papers reporting the same study)

Author (year); country	Study design; sample size and characteristics	Follow-up schedule	Intervention (breastfeeding training programme for midwives); control group	Outcome(s) and measure(s)	Results
Zakarija-Grkovic et al. (2012); Croatia [30]	Quasi-experimental design Intervention group: 385 mothers Control group: 388 mothers Mother/infant pairs were followed over a 12-month period both before and after training of maternity staff	Baseline and 3, 6, and 12 months postpartum	Intervention group: Programme: the UNICEF/WHO 20-h course Content: breastfeeding promotion and support Duration: 15.5 h of theory and 4.5 h of practice Providers: a neonatologist, a gynaecologist, a paediatrician, an economist/representative of a voluntary parenting group, a psychologist, and a general practitioner who is also a board-certified lactation consultant, midwife, and community nurse Materials: standard course materials including guidelines of course facilitators, outlines for course sessions, and PowerPoint slides for the course Control group: No training	Breastfeeding rate and initiation: self-designed questionnaires	• Compared with the control group, the proportion of newborns exclusively breastfed during the first 48 h after birth in the hospital was significantly higher in the intervention group (*p* < 0.01) • The proportion of mothers who initiated breastfeeding in the hospital in the control group was higher than that in the intervention group (*p* < 0.05) • No significant differences were seen in breastfeeding rates at 3, 6, or 12 months postpartum between the two groups
Ekström et al. (2012); Sweden [31]	RCT Intervention group (IG): 206 mothers Control group A (CGA; data were collected before the study period): 162 mothers Control group B (CGB; data were collected simultaneously with the intervention group): 172 mothers	Baseline, 3 days, 3 months, and 9 months postpartum	Intervention group: Programme: a process-oriented training programme Format: lectures and discussion Content: lectures on breastfeeding management and promotion, including discussions about counselling skills and attitudes, and reflection on personal breastfeeding experience Duration: 7 days Control group: No training	Breastfeeding duration and introduction of breastmilk substitutes: self-designed questionnaires and birth records	• IG mothers had a significantly longer duration of exclusive breastfeeding than the CGA mothers (*p* < 0.05) • Fewer IG infants received breast milk substitutes in the first week of life without medical reasons compared with CGA and CGB (*p* < 0.05) • IG infants were significantly older (3.8 months) when breast milk substitutes were introduced after discharge from the hospital compared with CGA and CGB infants (CGA = 2.3 months, *p* < 0.05; CGB = 2.5 months, *p* < 0.05)
Blixt et al.(2014); Sweden [32]				Breastfeeding problems and counselling satisfaction: self-designed questionnaires	• Among mothers with an exclusive breastfeeding duration < 3 months, IG mothers were more satisfied with the breastfeeding counselling provided (*p* < 0.01) and felt that the breastfeeding counselling was more coherent (*p* < 0.01) compared with CGA and CGB mothers • Fewer mothers with an exclusive breastfeeding duration < 3 months in the IG ended their breastfeeding due to insufficient breast milk supply compared with CGA and CGB (*p* < 0.05)
Ekström; et al.(2015); Sweden [33]				Breastfeeding initiation, duration, challenges, and introduction of breast-milk substitutes: self-designed questionnaires	• IG mothers reported earlier initiation (within 24 h), higher frequency (within 24 h), and longer duration of breastfeeding compared with CGA and CGB mothers (*p* < 0.05) • Less use of breast-milk substitutes in the first week of life without medical reasons and later introduction of breast milk substitutes after discharge from the hospital were reported by IG mothers compared with CGA and CGB mothers (*p* < 0.05) • IG mothers reported fewer breastfeeding challenges (e.g. insufficiency in breast-milk) than CGA and CGB mothers (*p* < 0.05)
Shamim et al. (2017); Bangladesh [34]	Pragmatic cluster RCT Baseline (pre-test): Control group (CG; provided services by midwives with no training): 461 mothers Intervention group (IG; provided services by midwives with breastfeeding training): 400 mothers Supervised group (SG, provided services by midwives with breastfeeding training and supervision): 321mothers Post-test: CG: 437 mothers IG: 358 mothers SG: 353 mothers	A pragmatic cluster RCT (baseline and 6 months after training)	Intervention group: Programme: breastfeeding training Theories/models: 2 separate training modules were developed by modifying the existing WHO/United Nations Children’s Fund 5-day breastfeeding counselling training guidelines Format: group facilitation, role plays, case studies, group work, demonstrations, field trips, and problem-solving discussions, Content: breastfeeding support Duration: 5 days Control group: No training	Self-designed questionnaires	• After training, the IG and SG had significantly higher proportions of mothers who reported early initiation of breastfeeding and avoidance of prelacteal feeds compared with the CG (*p* < 0.05) • No significant differences were seen in outcomes between the IG and SG • After training, exclusive breastfeeding rate was not significantly different among the groups

#### Study design

Of the 10 articles included, nine were published in English and one in Chinese. They were published between 2000 and 2019. Five RCTs were included [25, 31–34]. One study was reported in three separate papers each reporting on different outcomes [31–33]. Five reports described quasi-experimental studies [26–30].

#### Study settings

The settings of these studies were varied, including large and small hospitals in urban and rural areas. Two studies, reported in four articles, were carried out in Sweden [25, 31–33], two in the United Kingdom [26, 27], one in the United States [29], one in Croatia [30], one in Bangladesh [34], and one in Taiwan [28].

#### Randomisation method

Each of the included studies assigned participants to intervention or control groups in different ways. Five studies assessed the KAP towards breastfeeding of 386 midwives and nurses (sample sizes ranged from 28 to 135) [25–29]. Of these studies, two recruited midwives only [26, 27], while the other three recruited both nurses and midwives [25, 28, 29].

Four studies were quasi-experimental studies [26–29]. Moran et al. collected data from four sites (A, B, C, and D) [26]. As site A did not implement the training course, midwives at site A were assigned to the control group. All midwives at site B attended the course and were thus assigned to the experimental group. Despite the course being delivered at sites C and D, only some of the midwives had attended the course at the time of data collection. Therefore, midwives from sites C and D were assigned to both control (those who had not yet taken the course) and intervention (those who had taken the course) groups. In the study by Law et al. 108 midwives received breastfeeding training and 27 final-year student midwives constituted a control group [27]. Wang and Ku and Al-Nuaimi et al. recruited midwives from two hospitals using convenience sampling [28, 29]. One hospital was selected as the intervention group and delivered a breastfeeding training course. The other hospital served as the control group. In contrast, Ekström et al. carried out an RCT [25]. Their sampling frame consisted of 10 municipalities that were paired based on size and breastfeeding duration. The municipalities were then pairwise randomised to either an intervention group or a control group.

An additional three studies, reported in five articles, measured breastfeeding initiation, rates, and duration and included 3,463 mothers cared for by midwives (sample size ranged from 480 to 2,330) [30–34]. Zakarija-Grkovic et al. recruited mother/infant pairs before and after breastfeeding training of the maternity staff [30]. In their study, the breastfeeding training course was conducted twice: in May 2008 and February 2009. The control group was recruited from February to May 2008, and the intervention group was recruited from April to August 2009. Shamim et al. carried out their study in Bangladesh, which is divided into 64 districts, 493 sub-districts, and nearly 4,500 unions [34]. They randomly selected three sub-districts from the five sub-districts constituting the Panchagarh district. Of the 26 unions in the three selected sub-districts, nine unions were randomly selected and randomised into three groups. Outcomes were then compared between mothers in a control group (CG), who lived in unions where services were provided by midwives with no training; those in the intervention group (IG), who were living in unions where services were provided by midwives with breastfeeding training; and mothers in a supervision group (SG), who were living in unions where services were provided by midwives with breastfeeding training and supervision. Ekström et al., Blixt et al., and Ekström and Stina reported on the same study [31–33]. In this study, ten municipalities in Sweden were randomised to either an intervention group or a control group. Midwives in the intervention group were given breastfeeding training, while midwives in the control group were not. Mothers in the intervention or control municipalities were assigned to the intervention or control group, respectively.

#### Evaluation methods

All included studies used multiple instruments to measure various outcomes at different data collection time points. Moran et al. and Law et al. assessed breastfeeding knowledge and skills using the Breastfeeding Support Skills Tool (BeSST), a questionnaire including 20 open and ten closed questions based on four video clips [26, 27]. In the validation study, the BeSST demonstrated excellent internal reliability (Cronbach's α = 0.89) and inter-rater reliability (intraclass correlation coefficient = 0.96) [26, 35]. Wang and Ku used the Breastfeeding Knowledge Scale to assess breastfeeding knowledge and practice, which comprised 62 questions, each with a correct answer [28]. Total scores ranged from 0 to 62. The Kuder-Richardson Formula 20 (KR-20) of the scale was 0.83. Al-Nuaimi et al. used two validated questionnaires developed by the American Academy of Pediatrics to collect data about midwives’ breastfeeding knowledge and practices, but they did not report the reliability and validity of the scale [29].

To evaluate attitudes towards breastfeeding among midwives, Wang and Ku used the nine-item Breastfeeding Attitude Scale, which is assessed using a six-point scale ranging from strongly disagree (1 point) to strongly agree (6 points) [28]. The higher the score, the more positive the midwife’s attitude towards breastfeeding. The Cronbach’s α of the scale was 0.78. Ekström et al. used the Breastfeeding Attitudes Instrument, consisting of four dimensions relating to breastfeeding (regulating, facilitating, disempowering, and antipathy) [25]. The Cronbach’s α of the scale was 0.51. The internal consistencies for each factor were 0.80 (regulating), 0.60 (facilitating), for 0.62 (disempowering), and 0.29 (antipathy) [25]. Al-Nuaimi et al. used a seven-item questionnaire, but they did not report the reliability and validity of the scale [29].

The three studies that were reported on in five articles used self-designed questionnaires to test the secondary outcomes of breastfeeding initiation, rates, and duration [30–34]. Among them, Blixt et al. and Ekström et al. also collected data from maternal and/or neonatal medical records [31, 32].

### Methodological quality of the included studies

The methodological quality of the included studies is summarised in Tables 3 and 4. The overall quality of the included studies was moderate. The overall inter-rater agreement between the two independent reviewers of quality was good (kappa statistic = 0.80).

**Table 3:** Critical appraisal of the included randomized controlled trials (*n* = 5)

Appraisal questions	Ekström, Widstrom, & Nissen, 2005 [25]	Ekström, Kylberg, & Nissen, 2012 [31]	Blixt, Martensson, & Ekstrom, 2014 [32]	Ekström & Stina, 2015 [33]	Shamim, Dina, Vitta, & Greiner, 2017 [34]
1. Was true randomization used for assignment of participants to treatment groups?	Y	Y	Y	Y	Y
2. Was allocation to treatment groups concealed?	N	Y	Y	Y	Y
3. Were treatment groups similar at the baseline?	Y	Y	Y	Y	Y
4. Were participants blind to treatment assignment?	N	Y	Y	Y	Y
5. Were those delivering treatment blind to treatment assignment?	N	N	N	N	N
6. Were outcomes assessors blind to treatment assignment?	N/unclear	N/unclear	N/unclear	N/unclear	Y
7. Were treatment groups treated identically other than the intervention of interest?	N/unclear	N/unclear	N/unclear	N/unclear	N/unclear
8. Was follow up complete and if not, were differences between groups in terms of their follow up adequately described and analyzed?	Y	Y	Y	Y	Y
9. Were participants analyzed in the groups to which they were randomized?	Y	Y	Y	Y	Y
10. Were outcomes measured in the same way for treatment groups?	Y	Y	Y	Y	Y
11. Were outcomes measured in a reliable way?	Y	Y	Y	Y	Y
12. Was appropriate statistical analysis used?	Y	Y	Y	Y	Y
13. Was the trial design appropriate, and any deviations from the standard RCT design (individual randomization, parallel groups) accounted for in the conduct and analysis of the trial?	Y	Y	Y	Y	Y

**Table 4:** Critical appraisal of the included quasi-experimental studies (*n* = 5)

Appraisal questions	Moran, Bramwell, Dykes, & Dinwoodie, 2000 [26]	Law, Dunn, Wallace, & Inch, 2007 [27]	Zakarija-Grkovic et al., 2012 [30]	Hsiu-Ho Wang & Chieh-Yi Ku, 2012 [28]	Al-Nuaimi, Ali, & Ali, 2019 [29]
1. Is it clear in the study what is the ‘cause’ and what is the ‘effect’ (i.e. there is no confusion about which variable comes first)?	Y	Y	Y	Y	Y
2. Were the participants included in any comparisons similar?	Y	Y	Y	Y	N/unclear
3. Were the participants included in any comparisons receiving similar treatment/care, other than the exposure or intervention of interest?	N/unclear	N	N/unclear	N	Y
4. Was there a control group?	Y	Y	Y	Y	Y
5. Were there multiple measurements of the outcome both pre and post the intervention/exposure?	Y	Y	Y	Y	Y
6. Was follow up complete and if not, were differences between groups in terms of their follow up adequately described and analyzed?	Y	Y	Y	Y	Y
7. Were the outcomes of participants included in any comparisons measured in the same way?	Y	Y	Y	Y	Y
8. Were outcomes measured in a reliable way?	Y	Y	Y	Y	Y
9. Was appropriate statistical analysis used?	Y	Y	Y	Y	Y

Five studies assessed the KAP of midwives towards breastfeeding [25–29]. Most of these studies did not describe whether midwives received other breastfeeding training in addition to the intervention during the study period. Only two studies reported whether midwives had received prior breastfeeding training and evaluated the effects of this additional training on baseline levels of knowledge and compared the training rates between the groups [27, 28]. A quasi-experimental study by Al-Nuaimi et al. excluded nurses and midwives who had attended breastfeeding workshops or received prior training [29]. As the intervention is the breastfeeding training programme, midwives were aware of their study group allocation. Thus, the blinding of midwives was not possible, potentially leading to performance bias. Only one quasi-experimental study by Al-Nuaimi et al. provided the control group with an equal length training workshop on child growth and development from birth to five years of age [29].

The remaining five articles, reporting on the same three studies, assessed the initiation, duration, and rates of breastfeeding among postnatal mothers cared for by midwives [30–34]. Shamim et al. stated that in their RCT, both participating mothers and survey interviewers did not know which study group they were assigned to [34]. The other three articles, all reporting on the same study, similarly reported that participating mothers did not know if their midwife had attended the training programme, but they did not report if outcomes assessors were blinded to their group assignments [31–33].

For all 10 included articles, detection bias may also be present due to the subjective, self-reported nature of the evaluation of midwives’ breastfeeding attitude, mothers’ perception of professional support, and mothers’ satisfaction with the breastfeeding counselling. No participant dropout was reported during the study period by Al-Nuaimi et al., Law et al., and Wang and Ku, making the risk of attrition bias due to missing data low [27–29]. In contrast, five articles reported and compared the response rates of the participants in groups [25, 30–33]. An additional two studies were experimental studies based on repeated cross-sectional surveys, wherein comparisons were made between two groups of participants [26, 34].

### Effects of breastfeeding training programme

#### Primary outcome (midwives’ KAP towards breastfeeding)

**Fig. 2:**
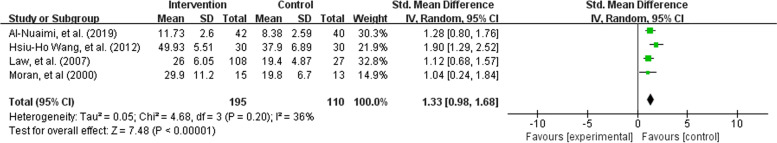
Meta-analysis of breastfeeding knowledge and skills

Of the five studies included in this review that measured the primary outcome, four measured the effects of a breastfeeding training programme on the knowledge and skills of midwives, with all reporting a positive effect (*p*< 0.05) [26–29]. The pooled results of these four studies (305 participants), combined as an SMD, was 1.33 (95%CI, 0.98 to 1.68) suggesting statistically significant beneficial effects (*p* < 0.01) of the breastfeeding training programme, with moderate heterogeneity (I^2^ = 36%) (See in Fig. 2).

Three studies examined changes in midwives’ attitudes, and all of these concluded that breastfeeding training programmes could improve their attitudes towards breastfeeding (*p*< 0.05) [25, 28, 29]. However, these studies could not be combined for meta-analysis due to heterogeneity of scales and scoring methods.

#### Secondary outcomes (breastfeeding initiation, duration, and rates among postnatal mothers)

The secondary outcomes were measured in three studies reported in five different articles [30–34]. Three studies assessed breastfeeding initiation [30, 33, 34]. One study reported that mothers in the intervention group reported earlier initiation (within 24 h after delivery) and higher frequency (within 24 h) of breastfeeding compared with mothers in the control group (*p*< 0.05) [33]. Similarly, Shamim et al. reported that the intervention group had a statistically significantly greater proportion of mothers reporting breastfeeding initiation ≤ 1 h after birth (340/353, 96.3%) compared with the control group (383/437, 87.6%) (*p*< 0.05) [34]. In contrast, Zakarija-Grkovic et al. reported that the proportion of mothers who initiated breastfeeding in the control group (387/388, 99.7%) was higher than that in the intervention group (378/385, 98.2%) (*p*< 0.05) [30]. However, meta-analysis of these three studies was not possible due to high heterogeneity (I^2^ = 98%).

The rates of exclusive breastfeeding, assessed by two included studies [30, 34]. Zakarija-Grkovic et al. reported that the proportion of newborns exclusively breastfed during the first 48 h after birth increased from 6.0% to 11.7% (*p*< 0.05) [30]. Unfortunately, this effect did not persist, with no differences seen in breastfeeding rates at discharge or at three, six, or 12 months postpartum between the groups. Similarly, Shamim et al. also reported no statistically significant difference in the rate of exclusive breastfeeding within six months between the groups [34].

Other benefits have also been reported: after breastfeeding training for midwives, mothers reported a statistically significantly longer duration of exclusive breastfeeding (*p*< 0.05) [31, 33], fewer breastfeeding challenges (*p*< 0.05) (e.g. insufficiency in breast-milk) [32, 33], higher satisfaction with the breastfeeding counselling received (*p*< 0.01) [32], and fewer infants receiving breast milk substitutes in the first week of life without medical reasons (*p*< 0.05) in the intervention group compared with the control group [31, 33, 34].

### Design of breastfeeding training programme

The design of the breastfeeding training programmes varied. Components, duration, materials, format, and provider were identified from the included studies.

#### Programme components

The breastfeeding training programmes in the included studies incorporated a range of different components. The three main components were breastfeeding theoretical knowledge, supportive skills, and counselling skills, which were incorporated by five included articles (describing three studies) in their breastfeeding training programmes [25, 29, 31–33]. The remaining five studies only included breastfeeding theoretical knowledge and supportive skills [26–28, 30, 34].

Breastfeeding training programmes containing all three components improved midwives’ knowledge [29], skills [29], and attitude towards breastfeeding [25, 29]. Additionally, such programmes were associated with the use of fewer breast milk substitutes in the first week of life without medical reasons, later introduction of milk substitutes after discharge from the hospital [31], earlier initiation of breastfeeding, higher frequency of breastfeeding, longer duration of breastfeeding, and fewer breastfeeding challenges [33], such as insufficient breast milk supply [32].

However, programmes that included only breastfeeding theoretical knowledge and supportive skills components were also associated with improved knowledge, skills, and attitude towards breastfeeding of midwives [26–28]. However, Zakarija-Grkovic et al. reported that the proportion of newborns exclusively breastfed during the first 48 h after birth increased, but there were no differences in breastfeeding rates at discharge or at three, six, or 12 months after birth between the intervention and control groups [30]. Shamim et al. similarly reported no statistically significant difference in the rate of exclusive breastfeeding between the groups [34].

#### Programme duration

The durations of the breastfeeding training programmes reported by the included studies varied from two hours to seven days. Of the ten included articles, Ekström et al., Ekström et al., Blixt et al., and Ekström and Stina had the longest training durations [25, 31–33]. They provided a seven-day process-oriented training programme for participants in the intervention group. Positive effects were reported for both primary and secondary outcomes. However, other long training durations (e.g. five days) led to higher proportions of mothers reporting early initiation of breastfeeding, but no statistically significant difference was observed in the rate of exclusive breastfeeding between the groups [34].

Two studies implemented the 20-h WHO/ UNICEF Breastfeeding Management Course and reported improved breastfeeding knowledge and skills among midwives and an increased proportion of newborns exclusively breastfed during the first 48 h after birth [26, 30]. However, no statistically significant differences were reported in breastfeeding rates at discharge or at three, six, or 12 months after birth between the intervention and control groups [30].

The remaining three studies provided midwives with short training sessions (two hours to eight hours) [27–29]. These studies reported positive effects on the KAP of midwives, but secondary outcomes were not assessed.

#### Teaching materials

Of the eight included studies, only four studies provided teaching materials to the midwives involved in the training programmes [26, 28–30]. Moran et al. provided midwives with a comprehensive, fully referenced workbook that could be used to cascade the training within their local maternity care services [26]. Zakarija-Grkovic et al. used standard course materials including guidelines for course facilitators, outlines for course sessions, and PowerPoint slides for the course [30]. The teaching materials used by Wang and Ku were the National Health Bureau, Department of Health, Executive Yuan Guidelines for Breastfeeding Teaching Materials in Taiwan and the Breastfeeding Question and Answer Manual [28]. Al-Nuaimi et al. developed two educational materials based on up-to-date evidence, including recommendations from the WHO and the National Institute for Health and Care Excellence (NICE), addressing the importance of breastfeeding initiation and child growth and development from birth to five years of age [29].

The remaining four studies, reported on in six articles, did not provide information about the teaching materials, if any were included in their programmes [25, 27, 31–34].

#### Programme format

Lectures and group discussions were the most common training programme formats and were used by three studies, reported on in five articles [25, 27, 31–33]. Role play was used by two studies [27, 34]. Additionally, group facilitation, group work, case studies, demonstrations, and field trips were also used in one training programme [34]. However, four studies did not provide any information about the format of the breastfeeding training programme implemented [26, 28–30]. All formats were found to be effective in improving both primary and secondary outcomes.

#### Training provider

Breastfeeding training programme providers varied widely among the included studies. The course provided by Moran et al. was organised in the UK by the UNICEF UK Baby Friendly Initiative Team and was taught by midwives and health visitors employed by UNICEF [26]. Wang and Ku invited breastfeeding teachers from the National Health Bureau, Department of Health, Executive Yuan to teach the course [28]. Shamim et al. organized a 5-day training course for the trainers by Training and Assistance for Health and Nutrition and Eminence [34]. The teachers of the course of Zakarija-Grkovic et al. were a neonatologist, a gynaecologist, a paediatrician, an economist/representative of a voluntary parenting group, a psychologist, and a general practitioner who was also a board-certified lactation consultant, midwife, and community nurse [30].

However, no details were provided in the six remaining articles regarding the characteristics of the person(s) providing the intervention in the four studies they reported [25, 27, 29, 31–33].

## Discussion

This systematic review evaluated the effects of breastfeeding training programmes for midwives on the primary outcome of the midwives’ KAP towards breastfeeding and the secondary outcomes of breastfeeding initiation, duration, and rates among postnatal mothers. It was found that the implementation of a breastfeeding training programme could improve midwives’ KAP. However, the breastfeeding training programmes had limited effects on breastfeeding initiation and rates.

Breastfeeding training is a requirement for maternity healthcare staff as stated in the BFHI. Thus, randomisation of midwives between receiving breastfeeding training and no training would contradict this basic requirement. As a result, few RCTs have been conducted for breastfeeding training programmes for midwives. Thus, it was deemed appropriate to include quasi-experimental studies in this review.

### The effects of breastfeeding training programmes on midwives’ KAP towards breastfeeding

We found that breastfeeding training programmes could improve midwives’ KAP, findings that align with previous systematic reviews [18, 19]. The studies included in this review were carried out in both developed and developing countries. This diversity suggests that the courses were effective in increasing midwives’ KAP towards breastfeeding, despite economic, ethnic, and cultural differences.

In most of the included studies, post-testing was conducted immediately or two weeks after the breastfeeding training, making it difficult to determine whether the associated changes persist in the long term [26–29]. Only one study measured the attitudes of midwives one year post training [25]. Moreover, factors such as policy changes and staff turnover may also affect long-term evaluation results. Therefore, to sustain the impact of such training programmes, regular in-service training is likely necessary.

### The effects of breastfeeding training programmes on breastfeeding initiation, duration, and rates among postnatal mothers

The definition of exclusive breastfeeding varied in five studies in terms of how secondary outcomes were measured [30–34]. Two studies followed the WHO definition of breastfeeding: ‘exclusive breastfeeding means no other food or drink, not even water, except breastmilk (including milk expressed or from a wet nurse) for the first six months of life, with the exception of rehydration solution (ORS), drops and syrups (vitamins, minerals and medicines)’ [30, 34]. In contrast, three studies used the definition of breastfeeding provided by the National Board of Health and Welfare, which was revised to align with the WHO definition of breastfeeding: ‘exclusive breastfeeding is breastfeeding with occasional use of water, breast milk substitutes (not more than a few times), and/or solids (not more than one tablespoon per day)’ [31–33].

Additionally, the length of the follow-up period also varied among the studies. Three studies reassessed the outcomes at three days, three months, and nine months postpartum [31–33], while one study followedup at three, six, and 12 months postpartum, or until discontinued [30]. In contrast Shamim et al. was a pragmatic clustered RCT with repeated cross-sectional surveys conducted six months apart [34]. Therefore, meta-analysis of these studies was not possible, and the results should be interpreted with caution.

Three studies assessed breastfeeding initiation [30, 33, 34]. Shamim et al. and Zakarija-Grkovic et al. reported opposite results relating to breastfeeding initiation rate [30, 34]. In terms of the rate of exclusive breastfeeding, both Shamim et al. and Zakarija-Grkovic et al. reported that it was not statistically significantly different between the intervention and control groups [30, 34]. This suggests that the breastfeeding training programmes had limited effects on breastfeeding initiation and rates. In a systematic review by Balogun et al., among all six studies included, only one examined the effects of breastfeeding training programmes for healthcare professionals on secondary outcomes and reported that the rate of exclusive breastfeeding increased [18]. However, no statistically significant differences were found in breastfeeding initiation rates, which differed from the results of the current review.

For other secondary outcomes, longer breastfeeding durations [31, 33], less and later introduction of breast milk substitutes without medical reasons [31, 33, 34], fewer breastfeeding challenges [32, 33], and higher maternal satisfaction were reported [32, 33]. This suggests that breastfeeding training programmes for midwives were effective in improving some breastfeeding outcomes.

### Breastfeeding training programme design

The results of this review suggest that the inclusion of counselling skills training, in addition to breastfeeding knowledge and skills training led to statistically significant positive effects on both primary and secondary outcomes. Besides, it was found that all training formats were effective in improving both primary and secondary outcomes.

Breastfeeding training programmes of different durations all resulted in increased KAP of midwives. Courses of longer duration correlated with more statistically significant effects on secondary outcomes [25, 31–33].

The course providers and teaching materials were often not reported. Despite this, the quality of the teachers and materials statistically significantly affected the effectiveness of the training programmes. More studies should be conducted to explore the effects of teachers’ characteristics (e.g. working years, experience, teaching ability) and teaching materials on breastfeeding training programme outcomes.

## Limitations

Some limitations of this review should be noted. First, the literature in this field is limited and all of the included studies had some methodological weaknesses. Second, in this review, the breastfeeding training programmes varied widely in terms of target audience, duration, content, providers, materials, and teaching methods. Thus, subgroup analyses to compare the effects of training duration, course contents, teaching methods, and teacher characteristics were not feasible. In addition to the heterogeneity of the training programmes, the measurement tools, assessment strategies, and outcome definition also varied, making meta-analysis not feasible for many outcomes. Lastly, only studies published in Chinese or English were included in this review, and expanding the analysis to other languages may provide additional evidence to support our conclusions.

## Conclusions

This systematic review has demonstrated that breastfeeding training programmes can improve midwives’ KAP towards breastfeeding. However, the breastfeeding training programmes had limited effects on breastfeeding initiation and rates.

More RCTs are required to explore the appropriate scientific content, methods, duration and provider of breastfeeding training for midwives, in addition to the effects of these variables on outcomes. Longitudinal studies are also warranted to examine the long-term effects of breastfeeding training programmes on midwives’ KAP, and breastfeeding initiation and rates towards breastfeeding. We suggest that future breastfeeding training programmes should incorporate counselling skills alongside breastfeeding knowledge and skills training.

## Data Availability

The datasets used and/or analysed during the current study available from the corresponding author on reasonable request.
